# Increasing cassava root yield on farmers' fields in Nigeria through appropriate weed management

**DOI:** 10.1016/j.cropro.2021.105810

**Published:** 2021-12

**Authors:** Friday Ekeleme, Alfred Dixon, Godwin Atser, Stefan Hauser, David Chikoye, Sam Korie, Adeyemi Olojede, Mary Agada, Patience M. Olorunmaiye

**Affiliations:** a International Institute of Tropical Agriculture, PMB 5320, Ibadan, Oyo State, Nigeria; b National Root Crops Research Institute, PMB 7006, Umuahia, Abia State, Nigeria; c Federal University of Agriculture, Makurdi, PMB 2373, Benue State, Nigeria; d Federal University of Agriculture, Abeokuta, PMB 2240, Ogun State, Nigeria

**Keywords:** On-farm trials, Herbicide, Manual hoe weeding, Cassava root yield, Net profit

## Abstract

Weed competition is the major biological stress affecting cassava production in smallholder farms in West and Central Africa, where yields are low compared with those in Asia and Latin America. Options for improved weed management are crucial in increasing productivity. Selected pre- and post-emergence herbicides, integrated with appropriate tillage and plant spacing, were tested in 96 sites in four locations in Nigeria, 24 in 2016 and 72 in 2017. Trials were split plots with six pre-emergence herbicides and no post-emergence treatment as main plots. Subplot treatments were four post-emergence herbicides, weeding with a motorized rotary weeder, short- and long-handled hoes, and no post-emergence weed control, i.e., regardless of pre-emergence treatments. Indaziflam-based treatments, irrespective of post-emergence treatment, and flumioxazin + pyroxasulfone applied pre-emergence followed by one weeding with a long-handled hoe provided >80% control of major broadleaf and grass weeds. Compared with herbicide use, farmer control practices (53%) were not efficient in controlling weeds. The highest root yield was produced where (1) s-metolachlor was combined with atrazine, and one weeding with a long-handled hoe or clethodim with lactofen, and (2) indaziflam + isoxaflutole was combined with glyphosate. An increase in root yield from 3.41 to 14.2 t ha-^1^ and from 3.0 to 11.99 t ha-^1^ was obtained where herbicides were used compared with farmers’ practice and manual hoe weeding. Our results showed that integrating good agronomic practices with safe and effective use of appropriate herbicides can result in root yield >20 t ha^−1.^ i.e., twice the national average root yield of 8–12 t ha^−1^, with >50% net profit. The use of appropriate herbicides can reduce the amount of manual labor required and improve livelihoods, specifically for women and children. Smallholder cassava farmers would require continuous training on the safe use and handling of herbicides to improve efficiency and prevent adverse effects on humans and the environment.

## Highlights

• Farmer control practices not efficient in controlling weeds compared to herbicide treatment.
• Herbicide use increased root yield 11–68% compared to nonherbicide treatments.
• Integrating good agronomic practices with herbicides leads to >50% net profit.
• Farmer weed control practice resulted in negative net profit.

## 1 Introduction

Cassava (*Manihot esculenta* Crantz) is a major staple food and affordable carbohydrate source for inhabitants of sub-Saharan Africa (Spencer and Ezedinma, 2017; Adiele, 2020). It is the most widely cultivated root crop in the tropics (Chandrasekara and Kumar, 2016; Titiek et al., 2017) because of its resilience to climate change, nutritional value, and use as a raw material for industrial products (Gleadow et al., 2016; Mupakati and Tanyanyiwa, 2017; Mtunguja et al., 2019). Nigeria is the global leader in cassava production: some 4.5 million farmers cultivate this crop over an area of 7.22 million ha with a yield exceeding 59 million t/yr (FAOSTAT, 2020). However, root yields in farmers' fields in Nigeria (8.20–12.22 t ha^−1^) are lower than those obtained in Asia and Brazil (FAOSTAT, 2020). For example, in Thailand, total production was 31.1 million t/yr, and the average root yield on 1.39 million ha of land was 22.4 t ha^−1^ in 2019.

Although many factors might account for the low productivity of farmers' fields, poor weed management was generally among the principal contributing factors. Initial slow growth makes cassava highly susceptible to the competition from weeds that reduces the number, size, and quality of the roots and starts (Ekeleme et al., 2016) in the early stage of crop growth before canopy closure. Early removal of weeds from the crop is, therefore, critical to achieving higher root yields. Weed control in the tropics is always a challenge and particularly for cassava cultivation. Farmers spend a large proportion of their resources on weed control, and the monetary cost associated with suppressing or controlling weeds exceeds the combined cost of controlling other pests (Chikoye et al., 2000). Earlier research revealed that weeding alone consumed approximately 30–50% of the total labor budget in the small-scale production systems typical of Nigerian agriculture, depending on the crop planted, type of weeds, and the level of other available resources (Akobundu, 1991; Jursik et al., 2011). Previous studies have shown that 50% of the yield reduction was caused by late and insufficient weeding (Chikoye et al., 2004; IITA, 2007). Tongglum et al. (2001) and Hauser and Ekeleme (2017) reported 25–100% reduction in root yield from uncontrolled weed interference. Similarly, Unamma et al. (1986) reported a decrease in root yield of 53 and 44% in two consecutive cropping seasons for cassava–maize intercrop from uncontrolled weed growth. Chikoye et al. (2005) reported that the absence of weed control resulted in yield losses of up to 100%.

Hand weeding is the predominant method employed by smallholder farmers. Consequently, they weed their fields three to four times, depending on the type of weeds and the extent of weed growth. Additional hoe weeding may be required where perennial weeds, such as *Imperata cylindrica* (L.) Raeusch. and *Panicum maximum* Jacq., dominate the population. Manual hand weeding is drudgery for farmers, particularly for the women who perform more than 90% of this task (Gianessi, 2010) – the drudgery either delays the work or prolongs the time needed for completion. The timely removal of weeds is an operation that needs improvement since root yield is affected by early competition. Melifonwu (1994) showed that the critical period for weed removal in cassava is 12 weeks after planting (WAP).

Chemical weeding, although dependent on certain factors such as its formulation and mode of action, the weed type, density, and the environment, offers an alternative to manual weeding because it is faster, less labour-intensive, and gives better control (Gianessi, 2010; Teasdale and Cavigelli, 2010). In West Africa, the use of herbicides by smallholder and medium-sized farmers is increasingly important for several reasons, including the increasing cost and widespread unavailability of the labor required to carry out traditional practices (Akobundu, 1980). Earlier studies of herbicide use showed higher root yields and lower net costs. For example, Chikoye et al. (2006) found that herbicide weed control in cassava was 30–50% cheaper than three manual weedings in southeastern Nigeria. In the same agroecological zone, Chikoye et al. (2007) reported a 54–96% reduction in labor costs from using herbicides to replace manual hoe weeding. Santigo et al. (2020) noted that pre-emergence herbicides decreased the need for manual weeding in cassava in Brazil. In Nigeria, the most popular herbicides for this purpose are atrazine, s-metolachlor + atrazine, glyphosate, and paraquat. Farmers apply s-metolachlor + atrazine immediately after planting and before the crop and weeds emerge.

In contrast, glyphosate and paraquat are used for land preparation to control the initial vegetation and sometimes as a directed spray under the canopy. Cassava is a long-duration crop, and the existing pre-emergence herbicides do not provide season-long control. Thus, they need to be supplemented with one or two hoe weedings or post-emergence herbicides applied directly to the weeds (Ekeleme et al., 2020). There is, therefore, the need to evaluate new pre- and post-emergence herbicides for season-long control in sole and intercropped cassava.

Although herbicide use to control weeds in cassava is generally increasing among farmers in Nigeria, only a limited number of pre- and post-emergence herbicides are registered for this purpose. Currently, diuron, atrazine and formulations containing atrazine and s-metolachlor are registered pre-emergence herbicides in cassava. No selective herbicides except fluazifop-p-butyl to control grass weeds are registered here specifically for post-emergence weed control in cassava fields. Multilocational trials were carried out in 2016 and 2017 to assess the efficacy of six selected pre-emergence and four post-emergence herbicides and three non-herbicide treatments on weeds and the response from root yield in on-farm cassava monocrops in Nigeria.

## 2 Material and methods

### 2.1 Study sites

**Fig. 1:**
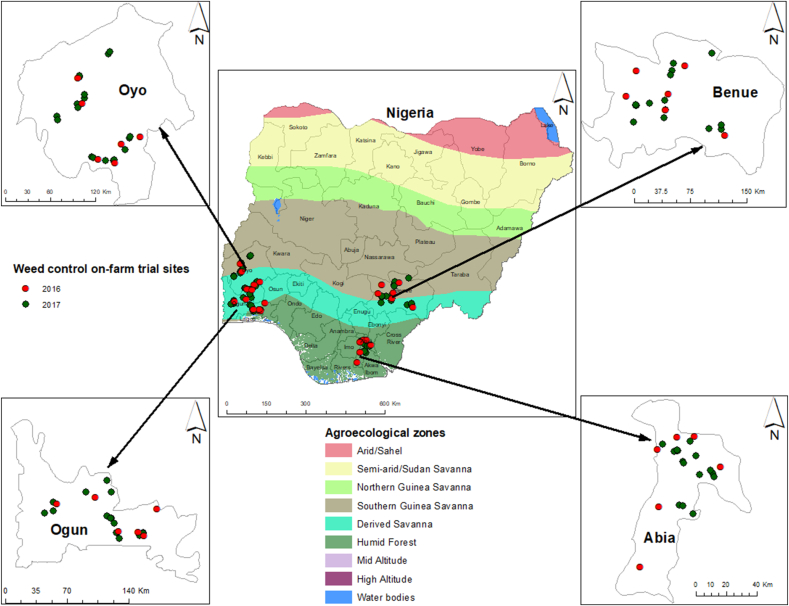
The study sites in Nigeria: Abia, Benue, Ogun, and Oyo States.

The trial was conducted at several sites in Abia, Benue, Ogun, and Oyo, major cassava producing States in Nigeria, from now onwards referred to as locations (Fig. 1). The trials were conducted in collaboration with the National Root Crops Research Institute (NRCRI) in Abia, Federal University of Agriculture Abeokuta (FUNAAB) in Ogun, Federal University of Agriculture Makurdi (FUAM) in Benue, and the Agricultural Development Authorities in the four States. Abia is in the Humid Forest (HF) agroecological zone. In Ogun majority of the sites are located in the Derived Savanna (DS), with some in the HF. Sites in Benue and Oyo are shared between the DS and Southern Guinea Savanna (SGS). The trials were set up in 24 farmers' fields in 2016 and 72 fields in 2017. At each location, farmers were selected from communities where an earlier baseline study on Knowledge, Attitudes, and Practices was conducted. Participating farmers donated land and committed themselves to protect the trials. Average annual rainfall at the sites in both years was 2081—2,184 mm in Abia, 1,287—1,605 mm in Benue, 1,209—1,577 mm in Ogun, and 1,119—1,262 mm in Oyo. At 0–20 cm depth, soil pH ranged from 5.18 to 5.31 in Abia, 5.63–5.86 in Benue, 5.49–5.88 in Ogun, and 6.04–6.27 in Oyo. The soil at the sites in the four locations varied from sandy clay loam to sandy loam with low N content [Abia, 0.13–0.14%; Benue, 0.5–0.09%; Ogun, 0.10–0.12% and Oyo, 0.07–0.10%]. Soil K at the various sites ranged from 0.27 to 0.31 cmol kg^−1^ in Abia, 0.24–0.27 cmol kg^−1^ in Benue, 0.27–0.31 cmol kg^−1^ in Ogun, and 0.25–0.32 cmol kg^−1^ in Oyo. Soil P was 9.13–11.17 mg kg^−1^ in Abia, 5.13–9.40 mg kg^−1^ in Benue, 7.13–9.03 mg kg^−1^ in Ogun and 6.53–11.07 mg kg^−1^ in Oyo. Before the trials were set up, existing vegetation at the trial sites was dominated by *I. cylindrica*, *P. maximum*, *Hyptis su*a*veolens* (L.) Poit., *Vernoniastrum ambiguum* (Kotschy and Peyr.) H.Rob., *Rottboellia cochinchinensis* (Lour.) Clayton, and *Passiflora foetida* L. Glyphosate was applied to existing weeds at 2000 g a.i. ha^−1^ 2 weeks before tillage.

### 2.2 Experimental design and treatments

In both years, the trials were set up as split plots in a randomized block design (Gomez and Gomez, 1984). In 2016, the main plots (pre-emergence weed management) had six herbicides and one plot had none. The subplots (post-emergence weed management) had four herbicides; mechanical weeding with a motorized rotary weeder, manual weeding with short- and long-handled hoes, and one plot had no post-emergence treatment. In order words, every main plot had a subplot without post-emergence treatment. The subplot in the no pre-emergence main plot that did not receive post-emergence treatment was used as a reference plot for the weed control efficacy rating. The size of each main plot was 7 × 71.6 m with 2 m alleys between the main plot and subplot. The trials were replicated in six farmers’ fields at each location. A farmer-managed plot measuring 7 × 57.6 m was installed beside the trial at each site. The chemical and nonchemical treatments for both plots are shown in Table 1, together with their respective commercial product names. In 2017, the plot without pre-emergence treatment was removed from the main plots; treatments with the use of long-handled hoe and motorized rotary weeder and those without post-emergence weed management were dropped from the subplots. Each pre-emergence herbicide in the main plots was assigned to three farmers randomly as replicates. The main plot size was 10 × 60 m with 2 m alley between subplots. At each site, a farmer-managed plot measuring 10 × 10.4 m was included as a control. A nontreated plot was also installed at each site as a reference plot for weed control efficacy rating. Farmer-managed plots were those where farmers controlled weeds using their methods of practice. Farmers methods of weed control varied from manual hoe weeding to a combination of manual hoe weeding with herbicides such as atrazine, diuron, glyphosate and paraquat in some sites. The only difference between the farmer-managed plot and the treatments evaluated was the method of weed control. The same crop variety and method of land preparation were used.

**Table 1:** Herbicides and application rates and other non-chemical post-emergence treatments evaluated on-farm in 2016 and 2017 in Nigeria for weed control in cassava.

Active constituents	Abbreviations	Application rate d(g a.i. ha^−1^)	Commercial mixtures
**Pre-emergence**			
Diflufenican + flufenacet + flurtamone	DIFF	135 + 360 + 180	Movon 450 SC^b^
Flumioxazin + pyroxasulfone	FLUP	110 + 140	Fierce 75 WG^c^
Indaziflam + isoxaflutole	INDI	75 + 225	Merlin Total 600 SC^b^
Indaziflam + metribuzin	INDM	56.3 + 720	Sencor Plus 517.5 SC^b^
S-metolachlor + atrazine	SMEA	1160 + 1480	Primextra Gold 600 SC^a^
S-metolachlor + terbuthylazine	SMET	1562.5 + 937.5	Gardoprim Plus Gold 500 SC^a^
No pre-emergence	NOPR	NA	NA

**Post-emergence**			
Clethodim + Lactofen	CLEL	0.147 + 0.128	Select Max + Cobra
Fluazifop-p-butyl + Lactofen	FLUL	450 + 0.128	Fusilade Forte + Cobra
Foramsulfuron-sodium + iodosulfuron-methyl-sodium + thiencarbazone-methyl + cyprosulfamide	FORT	47.2 + 1.6 + 15.0 + 22.5	MaisTer Power 57.5 OD^b^
Glyphosate	GLYP	2000	Touchdown Forte 500 SL^a^
No post-emergence	NOPS	NA	NA

**Non-herbicide treatments**			
Motorized rotary weeder	MOTO	NA	NA
Long-handled hoe	LOHH	NA	NA
Short-handled hoe	SHHH	NA	NA

a Supplied by Syngenta Crop Protection AG, Basel, Switzerland, https://www4.syngenta.com.

b Supplied by Bayer CropScience, Alfred-Nobel-Str. 50, Monheim, https://www.cropscience.bayer.com/en.

c Supplied by Valent USA Corporation, Walnut Creek, CA 94596, https://www.valent.com.

d g a.i. ha^−1^ = Active ingredients applied in grams per ha. NA = Not applicable.

In both years, 25 cm stem cuttings (variety TME 419) were planted at a density of 12,500 plants ha^−1^ and at an angle of approximately 45^O^ on ridges at a spacing of 100 × 80 cm. Pre-emergence herbicides were applied immediately after cassava was planted with a hand-pumped CP 15 (COOPER PEGLER®) knapsack sprayer calibrated to deliver 250 L ha^−1^ water at 240 kPa through a TEEJET XP 110–03 -VP nozzle. Post-emergence weed management treatments were conducted in the subplots at 8 WAP. Post-emergence herbicides were applied directly to weeds under the cassava canopy with a knapsack fitted with a blue flat fan (HARDI-ISO, F – 03–110) and hood to protect the crop. In 2016, where pre-emergence herbicides were not used, control was by one-time application of a post-emergence herbicide or three weedings at 4, 8, and 12 WAP using a motorized rotary weeder and short- and long-handled hoes. In 2017, manual hoe weeding with the short-handled hoe was conducted at 4, 8 and 12 WAP. Manual weeding with short-handled hoes at 4, 8, and 12 WAP is the current recommended time and frequency for weed control during the first growth cycle in a cassava monocrop in the study area. Each plot was assessed visually for control efficacy at 4, 6, 8, 10, and 12 WAP on a rating scale of 0–100% where 0 = no weed control, 10–49% = poor control, 50–69% = moderate control, 70–79% = fair/acceptable control, 80–89% = good control, and 90 to 100 = excellent control. Visual assessment at 10 and 12 WAP represents the efficacy rating of post-emergence treatments at 2 and 4 weeks after treatment.

Weed density was assessed at 4, 6, 8, 10, and 12 WAP by identifying and counting weed species in four 1-m^2^ quadrats placed randomly along two diagonal transects in each plot. Weed density assessment at 8 WAP was conducted before applying a post-emergence treatment. The cassava received 400 kg ha^−1^ of NPK 15:15:15 fertilizer at 4 WAP. Cassava establishment was assessed at 4 and 8 WAP in both years. Sprouted shoots were counted within net plots of 28 m^2^ in 2016, and 67.2 m^2^ in 2017 and data were converted to hectare. Roots were uprooted and harvested at 12 months after planting (MAP) from net plots of 28 m^2^ in 2016, and 67.2 m^2^ in 2017 and fresh stem weight was assessed. The storage roots were cut from the stem, cleaned of soil, and weighed for fresh root yield. Fresh stem weight was assessed at harvest. The stump, branches, and the top green part of the canopy were cut off, leaving a straight stem. For each net plot, the stems were tied in bundles of 50 and weighed on a hanging digital balance. Data were collected from sites in each location by field technicians and extension agents (EAs) in each location where the trials were conducted. Before the trials were set up, all field technicians and EAs from collaborating institutions and IITA had been trained on herbicide application and data collection using a Samsung Galaxy Tablet. Data collected from the various locations were transmitted to IITA and stored in the IITA Open Access/Open Data repository (CKAN). The research data preserved in IITA's CKAN were further extracted and analyzed.

### 2.3 Statistical analyses

Weed species density data at 8 WAP were used to estimate herbicide efficacy on major species as follows:

$$\frac{WSP_{untreated}-WSP_{treated}}{WSP_{untreated}}\times 100$$

Where *WSP*_*untreated*_ is the weed species population in untreated plots, and *WSP*_*treated*_ is the weed species population in treated plots. Analysis of variance was carried out on establishment, stem weight, stand population at crop harvest, fresh root yield, and herbicide efficacy using the Linear Mixed Model ANOVA procedure in SAS software (SAS 2013, version 9.4; SAS Institute Inc., Cary, NC) and separation of significant means was done using the SAS LSMEANS test (pair-wise *t*-test comparisons at P = 0.05). The data were analyzed by year as there were differences in the herbicide treatments regarding the numbers and setups used in the two trial years. In the ANOVA model, Location, Pre- and Post-emergence treatments, and their interactions were regarded as fixed effect factors, while Site (nested in Location) and Pre-emergence treatment interaction with Site (nested in Location) were considered as random effect factors, in consistence with the Split-Plot design used in the trial in multiple locations. A contrast statement was incorporated in the ANOVA model to obtain and test for the significant effect of the added control (farmer-practice plot) against the treated plots. Where two-way or higher interactions among main effects were significant (p < 0.05), simple effect differences were evaluated among treatments and represented with figures and graphics to understand the nature of the interactions. Counted data (cassava establishment and weed density) were log10(x+1) transformed before analysis to stabilize the variance and normalize the data (Gomez and Gomez, 1984). Pearson linear correlation coefficients were used to discern the level of association between (i) stem weight and weed density, (ii) root yield and weed density, and (iii) root yield and herbicide efficacy.

Cost-benefit analysis of the treatments evaluated was calculated using stem and root yields from the sites. The total cost of production (TCCP) for 1 ha was calculated as follows:

$$TCCP=x_{1i}\;+x_{2}\;+\cdot \cdot \cdot +x_{n}\;=\sum_{i=1}^{n}x_{i}$$

Where $x_{1}$ = Cost of pre-emergence herbicide, $x_{2}$ = Cost of post-emergence herbicide, $x_{3}$ = Cost of herbicide application, $x_{4}$ = Cost of land preparation, including ridging, $x_{5}$ = Cost of hoe weeding, $x_{6}$ = Cost of weeding with a motorized rotary weeder, $x_{7}$ = Cost of stems, $x_{8}$ = Cost of planting, $x_{9}=\text{ Cost of fertilizers},x_{10}$ = Cost of harvesting, $x_{n}$ = Cost of other *nth* items. Total Revenue (TR) was calculated for 1 ha as $TR=\;y_{1}+y_{2}=\overset{2}{\underset{i=1}{\sum }}y_{i}$ where $y_{1}$ = Revenue from stems$\text{ and}\;y_{2}$ = Revenue from roots. The net profit (NP) from production for 1 ha was calculated as the difference between TR and TCCP. In this analysis, the average minimum farmgate price/t of fresh roots was $27.4, and for one bundle of 50 stems, the price was $0.82. The selling price of fresh roots fluctuated between $27.4 and $95.8/t and stems between $0.82 and $2.74 for a bundle of 50. At the time of the analysis, 1 USD was equivalent to 365.5 Nigerian naira and 1 Euro to 436 naira.

## 3 Results and discussion

### 3.1 Cassava establishment and sensitivity of herbicides

Cassava establishment differed among locations at 4 and 8 WAP in 2016 (4WAP: F^3, 1294^ = 489.8, P = <0.0001; 8 WAP: F^2, 972^ = 426.0, P = <0.001) and in 2017 (4WAP: F^3, 390^ = 306.7, P = <0.0001; 8 WAP: F^3, 380^ = 426.0, P = <0.001). The interaction between pre-emergence treatments and locations was significant (Table 2). Cassava establishment was generally higher in Benue than in the other locations at 4 and 8 WAP, mainly due to differences in storage duration of stems at the different locations before planting. Stems were harvested from existing trials, preserved under shade for a maximum of 14 days, and transported to trial sites before they were cut into a 25 cm length for planting. Long storage durations have been reported to negatively affect cassava stake vigor and establishment (Boonma et al., 2007; Sungthongwises et al., 2016). The soil condition at planting at the different sites might also have contributed to differences in establishment among locations. Edaphoclimatic factors such as soil physical properties, moisture, and temperature are essential factors that can affect stem sprouting (Anikwe and Ikenganyia, 2018). When soil moisture is available, timeliness in planting operations is critical for healthy sprouting and good crop establishment (Anikwe and Ikenganyia, 2018). Within each location, establishment differed significantly among pre-emergence herbicide treatments at 4 and 8 WAP in both years except in Benue in 2017 (Table 2). Indaziflam-based herbicides caused a significant delay in sprouting at 4 weeks after treatment (WAT) in both years except at Benue and Ogun and at 8 WAT in Abia in 2017. Ekeleme et al. (2020) obtained similar results in another study in Oyo State in Nigeria and attributed the delay in sprouting to indaziflam, which suppressed buds exposed to the herbicide. Nicholas et al. (2019) reported that indaziflam at 155 and 234 g ha^−1^ caused transient stunting of Bermuda grass at 44 days after planting. Indaziflam is a nonselective cellulose-inhibiting herbicide that affects cell elongation and the growing meristematic regions (Brabham et al., 2014; Edwards et al., 2015; Neal, 2019).

**Table 2:** Effect of pre-emergence herbicide treatments and farmer practice on cassava establishment at 4 and 8 WAP in 2016 and 2017 in on-farm cassava monocrop sites at Abia, Benue, Ogun and Oyo States in Nigeria.

Pre-emergence treatments^a^	Abia	Benue	4WAP^b^	Oyo	Mean	Abia	Benue	8WAP	Oyo	Mean
			Ogun					Ogun		
	^_________________________^(%)^_________________________^					^________________________^(%)^_________________________^				
**2016**										
DIFF	72 aB	85 abA	73 bAB	58 aC	72 bc	NC	96 abA	84 abB	80 bB	87 bc
FLUP	67 bB	81 bA	81 abA	50 bC	70 c	NC	97 aA	86 aB	75 cdC	86 cd
INDI	51 dB	67 cA	64 cA	32 cC	53 e	NC	95 abA	76 cB	77 cB	83 e
INDM	58 cB	67 cAB	76 bA	36 cC	60 d	NC	94 bA	81 bB	74 cdB	83 e
SMEA	70 abB	86 aA	82 aA	59 aB	74 ab	NC	98 aA	87 aB	84 aB	90 a
SMET	71 abB	86 aA	83 aA	59 aC	75 a	NC	96 abA	87 aAB	83 abB	89 ab
NOPR	67 bB	89 aA	84 aA	56 aB	74 ab	NC	98 aA	84 abB	73 dC	85 d
*F-value*		5.34			104.60			5.52		15.7
*d.f.*		18, 1294			6, 1294			12, 972		6, 972
*P-value*		<0.0001			<0.0001			<0.0001		<0.0001

**2017**										
DIFF	55 dD	100 aA	76 bcC	85 bB	79 c	55 dC	100 aA	84 abB	91 abcB	83 c
FLUP	92 aA	100 aA	73 cB	54 dC	80 c	92 aB	100 aA	77 bcC	98 aAB	92 a
INDI	31 eD	100 aA	81 aB	59 dC	68 e	31 eC	100 aA	85 aB	90 bcB	77 d
INDM	53 dC	98 aA	75 bcB	68 cB	73 d	53 dC	98 aA	75 cB	94 abA	80 cd
SMEA	99 aA	99 aA	82 abB	86 abB	92 a	100 aA	100 aA	87 aB	83 cdB	92 a
SMET	72 cC	100 aA	84 aB	94 aA	88 ab	72 cC	100 aA	83 abB	93 abA	87 b
FPRA	84 bB	99 aA	82 abB	80 bB	86 b	81 bB	99 aA	86 aB	89 bB	89 ab
*F-value*		21.59			32.07			19.38		20.02
*d.f.*		18, 392			6, 392			18, 380		6,380
*P-value*		<0.0001			<0.0001			<0.0001		<0.0001

a Pre-emergence treatments are represented as DIFF, diflufenican + flufenacet + flurtamone; FLUP, flumioxazin + pyroxasulfone; INDI, indaziflam + isoxaflutole; INDM, indaziflam + metribuzin; SMEA, s-metolachlor + atrazine; SMET, s-metolachlor + terbuthylazine; NOPR, no pre-emergence; FPRA, farmer practice (data not collected in 2016).

b WAP = Weeks after planting; Different small letters within locations (Abia, Benue, Ogun, and Oyo) indicate significant differences in establishment among treatments at P < 0.05 probability level. Different uppercase letters indicate significant differences in establishment among locations for each pre-emergence treatment at P < 0.05 probability level. NC = Data were not collected.

### 3.2 Herbicide efficacy

Location had a significant effect on herbicide efficacy at all sampling periods in 2016 (4 WAP: F^3, 1186^ = 29.9, P = <0.0001; 6 WAP: F^2, 697^ = 14.7, P = <0.0001; 8 WAP: F^3, 1296^ = 189.4, P = <0.0001) and in 2017 (4 WAP: F^3, 326^ = 53.0, P = <0.0001; 6 WAP: F^3, 326^ = 6.25, P = 0.0004). The Location × Pre-emergence herbicide treatment effect on control was significant in both years (Table 3). In 2016, control was substantially lower where pre-emergence herbicide was not used, regardless of the location. All pre-emergence herbicides provided effective control (80–100%) at 4 WAP. Herbicide efficacy was better at Oyo at 6 WAP than in the other locations. At 8 WAP, indaziflam-based herbicides at Benue and Oyo and flumioxazin + pyroxasulfone at Benue provided better control than the others. In 2017, all pre-emergence herbicides provided effect control (80–100%) at all locations at 4 WAP, except for the diflufenican-based herbicide and s-metolachlor + atrazine at Oyo (Table 3). Flumioxazin + pyroxasulfone and s-metolachlor + atrazine in Ogun provided effective control up to 8 WAP in 2017. All herbicides provided superior control at all sampling times at all locations compared with farmer practice (Table 3). Overall, flumioxazin + pyroxasulfone, indaziflam + isoxaflutole, and indaziflam + metribuzin controlled major broadleaf weeds: *Ageratum conyzoides* L. (98–99%), *Aspilia africana* (Pers.) C. D. Adams (84–86%), *Chromolaena odorata* (L.) R.M. King and H. Rob. (76–80%), *Commelina diffusa* Burm. f. (90–100%), *Euphorbia heterophylla* L. (93–98%), *Mimosa invisa* Colla (78–85%), *Talinum triangulare* (Jacq.) Willd. (80–96%), *Tithonia diversifolia* (Hemsl.) A. Grey (95–98%), and *Tridax procumbens* L. (88–100%) without post-emergence treatment (data not shown). Major grass weeds controlled by flumioxazin + pyroxasulfone, indaziflam + isoxaflutole, and indaziflam + metribuzin were *Digitaria horizontalis* Willd. (82–100%), *Brachiaria lata* (Schumach.) C.E. Hubb. (81–82%), *P. maximum* (90–100%), and *Rottboellia cochinchinensis* (Lour.) Clayton (85–90%) (data are not shown). Earlier studies with these three herbicides in similar agroecology reported from very good (80–90%) to excellent (>90%) broadleaf control (Ekeleme et al., 2016, 2020), such as in *C. odorata* and *Calopogonium mucunoides* Desv.*,* and in grasses such as *P. maximum and R. cochinchinensis*. Sarangi et al. (2017) reported that flumioxazin + pyroxasulfone at 200 g a.i ha^−1^ provided up to 90% control of *Amaranthus tuberculatus* (Moq.) J.D. Sauer 21 days after application in Dodge County, Nebraska.

**Table 3:** Percentage of weeds controlled by pre-emergence herbicides and farmer practice at different locations (Abia, Benue, Ogun, and Oyo States) at 4, 6 and 8 weeks after treatment in on-farm trials in 2016 and 2017 in Nigeria.

Pre-emergence treatments^a^	Abia	Benue	4 WAP^b^	Oyo	Mean	Abia			Benue	6 WAP	Oyo	Mean		Abia		Benue			8WAP		Oyo			Mean
			Ogun							Ogun									Ogun					
**2016**	^_________________________________^(%)^_______________________________^					^__________________________________^(%)^_____________________________^								^__________________________________^(%)^___________________________^										
DIFF	88 bAB	92 bA	83 dB	89 cAB	88 d	79 aB			NC	71 cdC	85 bA	77 c		76 aAB		75 cAB			77 aA		66 bcdB			73 c
FLUP	91 abA	94 bA	89 bcA	91 bcA	91 c	79 aB			NC	74 bcB	90 abA	80 bc		78 aAB		81 bA			78 aAB		70 bB			77 b
INDI	96 aA	99 aA	94 aA	97 aA	96 a	81 aB			NC	84 aB	95 aA	85 a		75 aB		89 aA			78 aB		87 aAB			82 a
INDM	90 abB	100 aA	91 bB	94 abAB	94 b	78 aB			NC	78 abB	91 abA	81 b		79 aA		80 bcA			77 aA		86 aA			80 a
SMEA	93 aA	94 bA	88 cA	92 bA	92 c	80 aAB			NC	76 bcB	86 bA	80 bc		75 aA		79 bcA			77 aA		62 dB			73 c
SMET	90 abA	92 bA	89 bcA	92 bA	91 c	80 aB			NC	77 bB	90 abA	81 b		75 aA		78 bcA			77 aA		69 bcA			75 bc
NOPR	0 cA	0 cA	0 eA	0 dA	0 e	65 bA			NC	66 dA	38 cB	59 d		65 bB		79 bcA			59 bB		64 cdB			67 d
*F-value*			3.41		4175					11.53		52.0							6.67					23.58
*d.f*			18, 1186		6, 1186					12, 697		6, 697							18, 1296					6, 1296
*P-value*			<0.0001		<0.0001					<0.0001		<0.0001							<0.0001					<0.0001

**2017**																								
DIFF	85 abA	87 aA	90 abA	77 cB	85 b	81 aA			77 aA	77 cdA	64 cB	75 d		75 aA		77 aA			77 bc A		80 bA			77 b
FLUP	87 aB	93 aAB	90 abB	97 aA	92 a	85 aAB			82 aAB	90 aA	81 bB	84 a		77 aB		77 aB			81 bB		90 aB			81 a
INDI	88 aB	88 aB	92 aB	99 aA	92 a	77 aB			79 aB	85 abAB	89 aA	83 ab		77 aA		75 aA			77 bcA		80 bA			77 b
INDM	80 bB	90 aA	87 aA	89 bA	87 b	85 aA			75 aB	83 abcAB	79 bAB	81 abc		78 aAB		77 aAB			73 bcB		84 abA			78 ab
SMEA	85 abB	93 aA	87 aB	70 dC	84 b	83 aA			82 aA	69 deB	84 abA	79 bc		78 aB		75 aB			91 aA		78 bB			81 a
SMET	85 abA	87 aA	85 bA	82 cA	85 b	79 aA			77 aA	79 bcA	77 bA	78 cd		77 aB		77 aB			70 cB		87 abA			78 ab
FPRA	53 cB	45 bC	75 cA	32 eD	52 c	51 bB			36 bC	64 eA	41 dC	48 e		53 bB		62 bA			53 dB		43 cC			53 c
*F-value*		13.48			158				4.94			71.44					3.29					40.6		
*d.f*		18, 392			6, 392			18, 392					6, 392						18, 392				6, 392	
*P-value*		<0.0001			<0.0001			<0.0001					<0.0001						<0.0001				<0.0001	

a Pre-emergence treatments are represented as DIFF, diflufenican + flufenacet + flurtamone; FLUP, flumioxazin + pyroxasulfone; INDI, indaziflm + isoxaflutole; INDM, indaziflam + metribuzin; SMEA, s-metolachlor + atrazine; SMET, s-metolachlor + terbuthylazine; NOPR, no pre-emergence; FPRA, farmer practice.

b WAP = Weeks after planting; Different small letters within location (Abia, Benue, Ogun and Oyo) indicate significant differences in cassava establishment among treatments at P < 0.05 probability level. Different uppercase letters indicate significant differences in cassava establishment among locations for each treatment at P < 0.05 probability level. NC = Data were not collected.

At 12 WAP in 2016, the fixed effect factors (location, pre- and post-emergence treatments) and their interactions had a significant effect on control except for location × pre-emergence treatments (Table 4A). At all locations, control was least without post-emergence treatments (Abia, 59%; Benue, 63%; Ogun, 56%; Oyo, 42%) (data not shown). All post-emergence treatments had better control at Benue (83–88%) than at Abia (69–70%), Ogun (72–73%), and Oyo 56–65%), mainly due to the greater prevalence of perennial weeds at these locations than in Benue. Perennial weeds such as *T. diversifolia*, *P. maximum*, *M. invisa,* and *Commelina* spp. were more prevalent in Abia, Ogun, and Oyo. Pre- × post-emergence treatment effect on control was mainly due to the poor control where pre-emergence herbicides were not used (Table 5A). Diflufenican-based herbicide was the least effective compared with the other pre-emergence herbicides except when combined with the motorized rotary weeder, fluazifop-p-butyl, or foramsulfuron-sodium-based herbicide applied post-emergence (Table 5A). Except for post-emergence treatments, all fixed-effect factors did not affect control at 12 WAP in 2017 (Table 4A). Post-emergence control at 12 WAP ranged from 72 to 78%.

**Table 4:** Analysis of variance from mixed model procedures for (A) weed control efficiency (%) at 12 WAP, (B) cassava stand population at crop harvest, (C) cassava stem and (D) root yields as influenced by location and post-emergence treatment, their interactions and all treatments contrasted with farmer practice in 2016 and 2017 in on-farm trials in Nigeria.

		Variables											
		(A) Weed control efficiency (%) at 12 WAP			(B) Cassava stand population at crop harvest (no. ha^−1^)			(C) Cassava stem yield (kg ha^−1^)			(D) Cassava fresh roots yield (kg ha^−1^)		
Source of variation	df	DDF	F	Pr>F	DDF	F	Pr>F	DDF	F	Pr>F	DDF	F	Pr>F
**2016**													
Location (L)	3	19	8.16	0.0001	20	2.36	0.1025	19	6.8	0.0027	20	4.68	0.0124
Pre-emergence (Pr)	6	114	20.68	<.0001	969	0.96	0.4490	920	13.54	<.0001	969	21.34	<.0001
Post-emergence (Ps)	7	931	98.04	<.0001	969	5.67	<.0001	920	8.05	<.0001	969	13.18	<.0001
Pr × Ps	42	931	22.63	<.0001	969	1.85	0.0009	920	1.38	0.0553	969	2.62	<.0001
L × Pr	18	114	1.44	0.1283	969	0.1	1.0000	920	1.45	0.1005	969	1.02	0.4358
L × Ps	21	931	5.77	<.0001	969	2.03	0.0040	920	1.04	0.4078	969	2.49	0.0002
L × Pr × Ps	126	931	2.07	<.0001	969	1.27	0.0308	920	1.18	0.0984	969	1.07	0.2994
Treated^a^ vs Farmer's practice	1	NC	NC	NC	969	16.16	<0.0001	920	51.41	<0.0001	969	78.50	<0.0001

**2017**													
Location (L)	3	176	1.2	0.3124	111	0.280	0.8432	107	19.02	<.0001	111	3.53	0.0172
Preemergence (Pr)	5	184	0.52	0.7640	184	0.070	0.9963	176	1.38	0.2348	184	4.50	0.0007
Postemergence (Ps)	4	184	9.1	<.0001	184	2.690	0.0324	176	1.10	0.3562	184	1.57	0.1842
Pr × Ps	20	184	1.07	0.3857	184	0.390	0.9921	176	0.73	0.7873	184	1.68	0.0406
L × Pr	15	184	0.44	0.9658	184	0.040	1.0000	176	2.54	0.0020	184	0.80	0.6742
L × Ps	12	184	1.45	0.1486	184	0.710	0.7367	176	0.93	0.5146	184	1.31	0.2161
L × Pr × Ps	60	184	1.2	0.1805	184	0.740	0.9099	176	0.51	0.9985	184	0.88	0.7170
Treated vs Farmer's practice	1	184	45.43	<0.0001	184	3.13	0.0784	176	11.62	0.0008	184	37.34	<0.0001

a Treated refers to all pre- and post-emergence treatments. df, numerator degree of freedom; DDF, denominator degree of freedom of covariance parameter. The level of significance was set at P < 0.05 probability level. NC = Data were not collected in farmer practice plots in 2016.

**Table 5:** Percentage of weeds controlled on-farm in 2016 in Nigeria at 12 WAP (A), cassava stand population (B). and fresh root yield (C) as influenced by different treatment combinations and farmer practice.

Pre-emergence treatments^a^	Post-emergence treatments^b^							
	CLEL	FLUL	FORT	GLYP	LOHH	MOTO	NOPS	SHHH
(A) Weed control efficacy (%)								
DIFF	66 cC	74 bA	74 bA	68 cBC	67 cBC	70 bAB	57 cD	68 cBC
FLUP	76 bA	75 bA	76 bA	76 bA	86 aAB	73 bAB	72 bB	76 bA
INDI	83 aA	83 aA	84 aA	82 aA	84 aA	83 aA	80 aB	82 aA
INDM	80 aA	82 aA	80 aA	82 aA	80 aA	79 abA	80 aAB	81 aAB
SMEA	76 bA	75 bAB	74 bAB	74 bAB	73 bABC	69 cC	60 cD	71 bBC
SMET	74 bAB	73 bAB	76 bA	74 bAB	71 bB	73 bAB	62 cC	73 bAB
NOPR	61 cC	63 cBC	66 cAB	65 cAB	66 cAB	67 cdAB	0 dD	68 cA
(B) Cassava stand population (no. ha^−1^)								
DIFF	8938 bcAB	9573 aA	9431 aAB	7997 bcC	9096 bcAB	8707 bcBC	8850 aAB	8645 cdBC
FLUP	9583 abAB	8834 abBC	9256 aABC	8596 abBC	9376 abcAB	9810 aA	8500 aC	9387 abAB
INDI	8881 bcAB	9095 aAB	9075 abAB	8979 aAB	9193 bcdA	8770 bcAB	8399 aB	8973 bcAB
INDM	8854 bcAB	8986 aAB	8807 abAB	9184 aA	8566 cdAB	9154 bA	9143 aA	8292 cdB
SMEA	9967 aAB	9325 aBCD	9629 aABC	8818 abD	10112 aA	9497 abABCD	9043 aC	9659 abABC
SMET	9479 abAB	9451 aAB	9400 aAB	8366 abC	9953 abA	10015 aA	8826 aBC	10175 aA
NOPR	8053 cdA	7991 bcA	8177 bcA	6674 dB	8415 dA	7945 cdA	5882 bcB	7971 deA
FPRA	7282 d	7282 c	7282 c	7282 cd	7282 e	7282 d	7282 b	7282 e
(C) Cassava root yield (t ha^−1^)								
DIFF	22.89 abA	22.49 aA	22.67 aA	17.15 cC	21.32 bAB	21.08 abAB	19.08 aBC	20.72 abAB
FLUP	24.04 abA	23.06 aA	22.34 aAB	20.45 bB	20.49 bB	23.04 aA	19.86 aB	21.93 abAB
INDI	21.91 abAB	21.48 aAB	21.71 aAB	24.11 aA	22.16 bAB	21.72 abAB	18.71 aC	21.06 abBC
INDM	21.36 bA	20.25 aA	20.28 aA	21.71 abA	21.35 bA	19.36 bA	20.74 aA	20.24 bA
SMEA	24.53 aAB	22.63 aBCD	22.43 aBCD	21.76 abCD	25.82 aA	22.28 aBCD	20.42 aD	23.51 aABC
SMET	22.37 abAB	21.02 aBC	22.79 aAB	16.65 cD	22.30 bAB	23.91 aA	19.41 aC	22.73 abAB
NOPR	16.66 cA	16.57 bA	16.79 bA	12.18 dB	16.69 cA	15.23 cA	6.95 bC	15.79 cA
FPRA	10.56 d	10.56 c	10.56 c	10.56 d	10.56 d	10.56 d	10.56 b	10.56 d

a Pre-emergence treatments are represented as DIFF, diflufenican + flufenacet + flurtamone; FLUP, flumioxazin + pyroxasulfone; INDM, indaziflam + metribuzin; INDI, Indaziflam + isoxaflutole; SMEA, s-metolachlor + atrazine; SMET, s-metolachlor + terbuthylazine; NOPR, no pre-emergence; FPRA, farmer practice.

b Post-emergence treatments are represented as CLEL, clethodim + lactofen; FLUL, fluazifop-p-butyl + lactofen; FORT, foramsulfuron-sodium + iodosulfuron-methyl-sodium + thiencarbazone-methyl + cyprosulfamide; GLYP, glyphosate; LOHH, long-handled hoe; MOTO, motorized rotary weeder; NOPS, no post-emergence treatment; SHHH, short-handled hoe. Different small letters within each post-emergence treatment indicate significant differences treatment means at P < 0.05 probability level. Different uppercase letters indicate significant differences among post-emergence treatments at P < 0.05 probability level for each pre-emergence treatment.

Post-emergence herbicides were applied 8 weeks after pre-emergence herbicides. In plots where pre-emergence herbicides were not used, post-emergence herbicides were applied 4 weeks after planting cassava. Three weedings at 4, 8, and 12 WAP were carried out with either a motorized rotary weeder or short/long-handled hoes. Gianessi (2010) reported that manual hoe weeding with high labor demands is less effective than herbicides.

Indaziflam-based treatments, regardless of post-emergence treatment, and flumioxazin + pyroxasulfone applied pre-emergence and followed by one weeding with a long-handled hoe were the most effective in controlling weeds (Table 5A). These treatments provided 80–84% control at 12 WAP in 2016. Control with indaziflam-based herbicides and flumioxazin + pyroxasulfone at 12 WAP was as good as when these herbicides were supplemented with post-emergence treatments (Table 5A). This suggests these herbicides at the rate evaluated in this study may not require supplementary post-emergence control before canopy closure. Previous studies have reported long-season residual control from indaziflam + metribuzin, indazuflam + isoxaflutole, and flumioxazin + pyroxasulfone (Mahoney et al., 2014; McNaughton et al., 2014; González-Delgodo et al., 2015; Currie and Geier, 2016; Curtis et al., 2016; Jeschke, 2016; Ekeleme et al., 2016, 2020). Currie and Geier (2016) observed that indaziflam + metribuzin at 0.21 + 0.75 kg ha^−1^ and 0.35 + 0.75 kg ha^−1^ was the most effective in controlling *Bassia scoparia* (L.) A. J. Scott. (80%) when evaluated with other treatments in an abandoned *Medicago sativa* L. field. Indaziflam alone at label rate has been reported to provide >90 days of broad-spectrum control in citrus in Florida. Indaziflam + metribuzin is registered for control in perennial crops and flumioxazin + pyroxasulfone in soybean. Farmer control practices (53%) were not efficient in controlling weeds compared with herbicide use in 2017 (data not shown).

### 3.3 Cassava stand population at harvest

The numbers in the population at crop harvest did not differ among locations and pre-emergence herbicide treatments in both years (Table 5B). Location × pre-emergence herbicide treatment interaction did not affect the population in either year (P>0.05). Post-emergence herbicide treatments had a significant effect on the cassava population in both years. The population was lowest where glyphosate was applied post-emergence (8374 plants ha^−1^) in 2016 and in the nontreated plot in both years (2016: 8424 plants ha^−1^; 2017: 4732). Although glyphosate was applied post-emergence directed to weeds under the canopy, injuries were observed on some plants at some sites, which might have been caused by herbicide drift. Liu et al. (1982) reported crop injury with two post-emergence applications of glyphosate under the cassava canopy in Puerto Rico. Glyphosate is a non-selective herbicide. In 2017, the populations were comparable where fluazifop-p-butyl + lactofen (10990 plants ha^−1^), foramsulfuron-sodium-based herbicide (10773) and clethodim + lactofen (10786) were used post-emergence, but higher than in treatments with glyphosate (10390 plants ha^−1^) and the short-handled hoe (10484) (data not shown). Location × post-emergence treatment interaction had a significant impact on the population in 2016 but not in 2017 (Table 5B). Cassava population was lowest in Abia (5952–7039 plants ha^−1^) than in the other locations (Benue: 9184–10655, Ogun: 9535–10094, Oyo: 7959–9940) regardless of post-emergence treatment. Within locations, the populations were.

Similar among post-emergence treatments except for sites at Abia where the lowest population was observed in the nontreated plots and at Oyo in the glyphosate treated plots. Pre- × post-emergence treatment interaction had a significant effect on population in 2016 only (Table 4, Table 5B). The population was lowest where pre-emergence herbicide was not used except with indaziflam-based herbicides and flumioxazin + pyroxasulfone, indicating the importance of pre-emergence herbicide in eliminating early weed competition with the crop. Diflufenican-based herbicide combined with glyphosate and the no-pre-emergence herbicide treatments had the lowest populations compared with the other treatments.

### 3.4 Fresh cassava stem weight

**Fig. 2:**
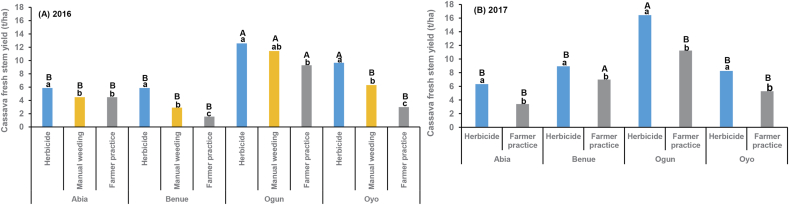
Fresh cassava stem yield (t ha^−1^) as affected by herbicide, manual hoe weeding, and farmers' practice treatments at four on-farm locations (Abia, Benue, Ogun, and Oyo States) in 2016 and 2017. Treatment bars within the same location with the same lowercase letters are not significantly different at α = 0.05. Across locations, the same treatment bars with the same uppercase letters are not significantly different at α = 0.05.

Location had a significant impact on the fresh stem weight in both years (Table 4C). Stem weight in treated plots ranged from 4.55 to 8.14 t ha^−1^ in Abia and was 3.07–15.75 in Benue, 10.52–25.33 in Ogun, and 5.51–12.50 in Oyo (data not shown). Ogun sites produced heavier stems in both years (2016: 10.72 t ha^−1^; 2017: 9.80) than the other locations where the average weight ranged from 3.52 to 5.89 t ha^−1^ in 2016 and from 3.85 to 6.53 in 2017 regardless of treatment (data not shown). Stem weight differed significantly among pre- and post-emergence treatments in 2016 (Table 4C). Averaged across locations and post-emergence treatments, plots treated with s-metolachlor + atrazine (9.09 t ha^−1^) produced heavier stems than the other treatments where stem weight ranged from 7.97 to 8.56 t ha^−1^. The farmer practice plots had the lowest stem weight [4.58 t ha^−1^] (data not shown). The contrast analysis of all herbicide treatments with manual hoe weeding and farmer practice showed significant differences in stem weight at all locations (Table 4C, Fig. 2). The stem weight in the herbicide treated plots was 1.3–3.8 times heavier than those in the farmer practice plots in 2016 and 1.3 to 1.9 times in 2017 (Fig. 2). Where herbicides were used, an increase in stem weight of 50% was observed in Benue and 35% in Oyo compared with the manual hoe weeding practice (Fig. 2A). Although not significant, stems tended to be heavier where herbicides were used at Abia and Ogun than those where manual hoe weeding was conducted. There was a significant negative relationship between stem weight and weed density at 8 WAP in both years (2016: r = −0.22, p = <0.0001, n = 1270; 2017: r = −0.28, p = <0.0001, n = 383) indicating that stem weight in the farmer practice plots was depressed by competition as weed density increased. Agahiu et al. (2011) reported a significant negative relationship (r = −0.796) between weed biomass and stem girth in Kogi State, Nigeria. Also, Alabi et al. (2001) reported a reduction of between 13 and 40% in stem girth in fields infested with *M. invisa* at Ibadan, Nigeria. There is a positive linear relationship between stem girth and weight.

### 3.5 Cassava root yield

Fresh root yield varied with location in both years (Table 4D). In 2016, root yield ranged from 4.06 to 30.88 t ha^−1^ in Abia and was 1.31–45.16 in Benue, 10.29–48.17 in Ogun, and 1.94–45.01 in Oyo (data not shown). In 2017, root yields ranged from 7.01 to 40.58 t ha^−1^ in Abia and were 3.72–39.82 in Benue, 5.61–47.65 in Ogun, and 1.94–30.55 in Oyo. Averaged across locations and post-emergence treatments, root yield was different among pre-emergence herbicide treatments in both years (Table 4D).

The two- and three-way interactions of location, pre-, and post-emergence treatments did not affect root yield in either year (Table 4D). Pre- × post-emergence treatment interaction positively impacted root yield in both years (Table 5, Table 6). In 2016, the highest root yield was produced where (1) s-metolachlor + atrazine was combined with one weeding with a long-handled hoe or with clethodim + lactofen and (2) indaziflam + isoxaflutole was combined with glyphosate. Root yields were depressed in plots treated with diflufenican-based herbicide or s-metolachlor + terbuthylazine combined with glyphosate used post-emergence. Root yields where pre-emergence herbicides were used were generally higher with post-emergence weed management than without it except with the following treatment combinations (1) all treatment combinations with indaziflam + metribuzin (2) flumioxazin + pyroxasulfone combined with foramsulfuron-sodium-based herbicide, glyphosate, long- or short-handled hoes (3) indaziflam + isoxaflutole combined with short-handled hoe (4) s-metolachlor + atrazine combined with fluazifop-p-butyl + lactofen, foramsulfuron-sodium-based herbicide, glyphosate or motorized rotary weeder, and (5) s-metolachlor + terbuthylazine combined with fluazifop-p-butyl + lactofen or glyphosate. The lowest root yields in 2016 were obtained from treatments where a pre-emergence herbicide was not applied and in the farmer practice plot. In 2017, the highest root yields were obtained from flumioxazin + pyroxasulfone or indaziflam + metribuzin combined with clethodim + lactofen or foramsulfuron-sodium-based herbicide or one manual hoe weeding with the short-handled hoe (Table 6).

**Table 6:** Cassava fresh root yield as influenced by different treatment combinations and farmer practice in on-farm trials in 2017 in Nigeria.

Pre-emergence treatments^a^	Post-emergence treatments^b^					
	CLEL	FLUL	FORT	GLYP	SHHH	Mean
	^____________________________________________^(t ha^−1^)^_______________________________________________^					
DIFF	14.72 cdA	15.14 bcA	14.13 cdAB	12.33 bcAB	11.07 cB	13.48 cd
FLUP	24.95 aA	22.93 aA	24.64 aA	22.57 aA	21.87 abA	23.39 a
INDI	19.86 abAB	21.31 aA	18.71 bcAB	17.98 abB	18.77 abAB	19.33 ab
INDM	24.83 abA	21.46 aB	22.39 abAB	21.30 aB	24.10 aAB	22.82 a
SMEA	13.37 dB	19.16 abA	16.74 bcAB	16.18 bcAB	16.27 bcAB	16.34 bc
SMET	19.34 bcA	15.26 bcB	17.64 bcAB	17.62 abAB	17.94 bAB	17.56 bc
FPRA	11.92 d	11.92 c	11.92 d	11.92 c	11.92 c	11.92 d

a Pre-emergence treatments are represented as DIFF, diflufenican + flufenacet + flurtamone; FLUP, flumioxazin + pyroxasulfone; INDM, indaziflam + metribuzin; INDI, Indaziflam + isoxaflutole; SMEA, s-metolachlor + atrazine; SMET, s-metolachlor + terbuthylazine; FPRA, farmer practice.

b Post-emergence treatments are represented as CLEL, clethodim + lactofen; FLUL, fluazifop-p-butyl + lactofen; FORT, foramsulfuron-sodium + iodosulfuron-methyl-sodium + thiencarbazone-methyl + cyprosulfamide; GLYP, glyphosate; SHHH, short-handled hoe; Different small letters within each post-emergence treatment indicate significant differences treatment means at P < 0.05 probability level. Different uppercase letters indicate significant differences among post-emergence treatments for each pre-emergence treatment at P < 0.05 probability level.

**Fig. 3:**
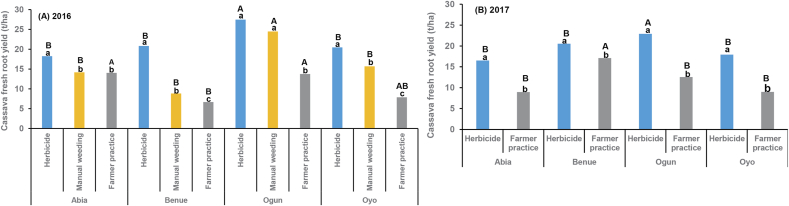
Fresh cassava root yield (t ha^−1^) as affected by herbicide, manual hoe weeding, and farmers' practice treatments at four on-farm locations (Abia, Benue, Ogun, and Oyo States) in 2016 and 2017. Treatment bars within the same location with the same lowercase letters are not significantly different at α = 0.05. Across locations, the same treatment bars with the same uppercase letters are not significantly different at α = 0.05.

The contrast between all herbicide treatments with manual weeding and farmer practice was significant for root yield at all the locations in both years (Table 4D, Fig. 3). In both years, the average root yield in herbicide and manual hoe weeding treatments was higher in Ogun than in the other locations (Fig. 3). An increase of 10.9–67.9% in 2016 (Abia: 4.2 t ha^−1^, Benue: 14.2 t ha^−1^, Ogun: 13.7 t ha^−1^, Oyo: 12.6 t ha^−1^) and of 15.2–49.8% (Abia: 7.6 t ha^−1^, Benue: 3.4 t ha^−1^, Ogun: 10.4 t ha^−1^, Oyo: 8.9 t ha^−1^) in 2017 was obtained across all locations where herbicides were used compared with farmer practice. In 2016, an increase in root yield of 4.08 t ha^−1^ in Abia, 11.99 t ha^−1^ in Benue, 3.0 t ha^−1^ in Ogun and 4.78 t ha^−1^ in Oyo was observed across locations where herbicides were used compared with the manual hoe weeding, indicating that the use of herbicides in cassava production was a better alternative (Fig. 3A). In this study, the manual hoe weeding was carried out at 4, 8, and 12 WAP during the first growth cycle of cassava. Although the recommended weeding periods and frequencies were followed precisely, the weeding at 4 WAP did not protect the crop from the early-season competition; some sites had weeds before 4 WAP, especially perennial weeds, which might have resulted in competition. Therefore, the current recommended manual weeding periods of 4, 8, and 12 WAP for cassava production need to be adjusted and made site-specific, based on the weed spectrum. Previous studies have stressed the importance of timely manual control in smallholder farms in enhancing root yield (Gianessi, 2010; Khanthavong et al., 2016; Kintché et al., 2017). For example, Kintché et al. (2017) reported that root yield was significantly higher by 2 t ha^−1^ in the Kongo Central and Tshopo provinces of the Democratic Republic of Congo when manual weeding was conducted the first month after planting than when carried out during the second month. Root yield where herbicides were used was greater by 10.9 to >100% in 2016 and by 15.2 to >90% in 2017 (Fig. 3) across locations mainly due to competition as a result of poor, untimely, and ineffective weeding. Gianessi (2010) reported that most smallholder farmers in Africa do not carry out enough weeding or weed at the right time and attributed this to labor availability and time constraints. Kintché et al. (2017) reported that late and sparse field weeding are vital factors limiting root yield. Studies by Sarangi et al. (2017) showed that early-season control could enhance crop yield. There was a significant positive correlation between herbicide efficacy and root yield (2016: r = 0.20, p = <0.0001, n = 1277; 2017: r = 0.55, p = <0.0001, n = 355) and also a significant negative correlation between weed density and root yield (2016: r −0.28, p = <0.0001, n = 1138; 2017: r = −0.44, p = <0.0001, n = 355) demonstrating a positive impact on root yield from early weed removal with herbicides and also the negative impact of increased weed pressure on root yield.

### 3.6 Cost-benefit analysis

**Fig. 4:**
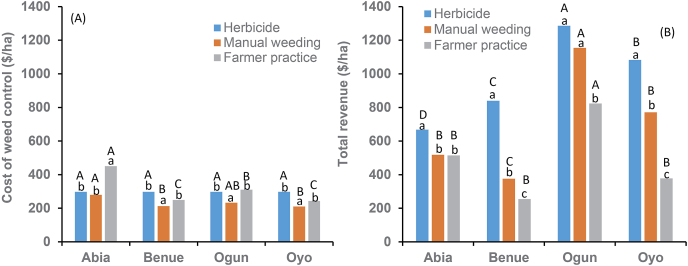
The average cost of weed control and the net profit in using herbicides, best hoe weeding, and farmer practice in 96 on-farm cassava mono-crop sites in Nigeria. The best hoe weeding practice refers to timely weeding at 4, 6, and 8 weeks after planting cassava.

**Fig. 5:**
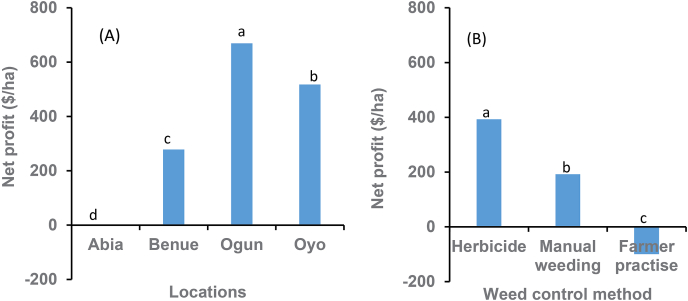
Net profit by locations (A) and weed control methods (B) in Nigeria. The manual weeding refers to timely hoeing weeding at 4, 6, and 8 weeks after planting cassava.

The result of the cost-benefit analysis showed that the cost of weed control varied within and across locations (Fig. 4). The farmers’ method of weed control was more expensive than manual hoe weeding. In this study, farmers weeded more than four times at most sites, depending on the weed pressure. The disparity in the cost of control was more pronounced in Abia than in the other locations. Abia is located in the humid forest zone characterized by high rainfall and relative humidity, which usually promotes high weed growth requiring frequent weeding. Adigun et al. (2018) reported that heavy rain and high relative humidity favored rapid and excessive weed growth in southwestern Nigeria. Except at Abia, the cost of control with herbicide was higher than the cost of four manual hoe weedings. Total revenue differed among control methods within and between locations (P =<0.0001; Fig. 4B). Total revenue was substantially higher where herbicides were used than either manual hoe weeding or farmer practice at all locations except at Ogun (Fig. 4B), where total revenue from manual weeding was comparable with revenues from herbicide treatment. Farmer practice generated the lowest revenue except at Abia. Net profit differed with location and control methods (P =<0.0001; Fig. 5). Regardless of the method, net profit was more substantial in Ogun than in the other locations (Fig. 5A). It was more profitable (51%) to control weeds with herbicides than manual weeding (Fig. 5B). A negative net profit was observed when farmers managed weeds in their plots using their usual control method, mainly due to the high cost and low root and stem yield.

Although net profit was higher where herbicides were used than in the best practice weeding, the total cost of control using herbicides was slightly higher, mainly because of the herbicides used in this study. Apart from s-metolachlor + atrazine, fluazifop-p-butyl, and glyphosate, all other herbicides were imported at the prevailing selling price overseas. When the cost of control using herbicides available in Nigeria (s-metolachlor + atrazine used pre-emergence combined with glyphosate used post-emergence) was compared with best practice manual weeding, no significant increase in the cost of control was observed. Chikoye et al. (2006) found that herbicide control was 30–50% less expensive than three manual hoe weedings in southeastern Nigeria.

The higher net profit from herbicides was attributed to differences in revenue from roots and stems due to higher yields. For example, root and stem yields were negatively correlated with weed density. These results were similar to Adinya et al. (2007), who reported a profit of $15.3 ha^-1^ in smallholder farmers' fields in Akwa-Ibom State, Nigeria, and labor for weeding consumed 63% of the total cost of production. Early weed removal was achieved with herbicides which eliminated competition with the crop at the early growth stages and enhanced crop yield.

## 4 Conclusion

Our results showed that indaziflam + isoxaflutole, indaziflam + metribuzin, and flumioxazin + pyroxasulfone provided 72–80% control of broadleaf and grassy weeds up 12 WAP without post-emergence weed control. Farmer control practices were not efficient in controlling weeds. Cassava stem yield increased by 9.2% more where herbicides were used than the manual hoe weeding and 50.3% more than in farmer practice. Similarly, cassava root yield where herbicides were used was 10.9–57.6% more than the manual hoe weeding plots. The lowest root yields were obtained from treatments where a pre-emergence herbicide was not applied and in the farmer practice plot. The net profit from the root and stem yields increased by 51%, using herbicides compared to manual hoe weeding. Overall, considering net profit; the following treatments were the most promising for weed control in cassava mono-crop: (in decreasing order of prominence) 1.) flumioxazin + pyroxasulfone combined with clethodim + lactofen or foramsulfuron-sodum based herbicide, 2.) indaziflam + metribuzin with clethodim + lactofen or one manual hoe weeding using the short-handled hoe, 3.) indaziflam + isoxaflutole combined with glyphosate, and 4.) s-metolachlor + atrazine combined with one weeding with a short-handled hoe. However, care must be taken in using indaziflam-based combinations since indaziflam delays sprouting, which might affect the population, especially with poor calibration. The increased yields and profits from herbicide use may lead to improved farmer livelihoods, specifically of the women and children who bear most of the burden of weed control in cassava. Using herbicides rather than weeding alone also reduces the amount of manual labor required to grow cassava.

## Declaration of competing interest

The authors declare that they have no known competing financial interests or personal relationships that could have appeared to influence the work reported in this paper.
